# Editorial: Recent Advances in Fluorescent Probes for Super-Resolution Microscopy

**DOI:** 10.3389/fchem.2021.698531

**Published:** 2021-06-11

**Authors:** Fan Wang, Xusan Yang, Qiuqiang Zhan, Chayan K. Nandi

**Affiliations:** ^1^ School of Electrical and Data Engineering, University of Technology Sydney, Sydney, NSW, Australia; ^2^ School of Applied and Engineering Physics, Cornell University, Ithaca, NY, United States; ^3^ South China Academy of Advanced Optoelectronics, South China Normal University, Guangzhou, China; ^4^ School of Basic Sciences, Indian Institute of Technology, Mandi, India; ^5^ Advanced Materials Research Centre, Indian Institute of Technology, Mandi, India; ^6^ BioX Centre, Indian Institute of Technology, Mandi, India

**Keywords:** super resolution, fluorescent probe, nanodots, localization microscopy, optical bioimaging

Super-resolution microscopy (SRM) has become an indispensable tool for monitoring cytoskeleton dynamics as well as the imaging, detection, and tracing of functional biomolecules in living cells. It overcomes the barrier of diffraction limit and allows for the visualization of sub-cellular structures down to the sub-10 nm level. Several types of SRM techniques are available, with a vast number of applications in interdisciplinary research fields. To date, many fluorescent probes such as fluorescent proteins, organic dyes, nanomaterials, quantum dots, polymer dots, organic dots, carbon dots, and upconversion nanomaterials have been employed for SRM. Each has its own limitations, and, as such, in order to obtain a better-resolved structure, improving the efficiency of these probes is of great importance.

The performance of fluorescent probes in super-resolution microscopy is dependent on several important photo-physical parameters. Organic molecules, with their smaller size, have excellent blinking properties, but they are often dim and undergo rapid photobleaching. Photoactivable or photoswitchable fluorescent proteins suffer from poor localization precision due to their low photon counts. Nanomaterials, quantum dots, carbon dots, polymers dots, etc. have shown promising results due to their high brightness and photostability, but their large size compared to organic molecules limits their potential applications.

In this research topic, we present a collection of original research, mini review, perspective, and review articles touching upon different aspects of new fluorescent probe materials that advance the SRM techniques one step ahead to the current state of the art in the field. We also provide a new labeling strategy of the fluorescent probe in the live cell SRM imaging.

Han et al., in their research article on “a labeling strategy for living specimens in long term super resolution fluorescence imaging”, showed the capability of many fluorescent dyes to stain various types of subcellular structures in living specimens with high brightness and photostability. They used several known dyes, such as Atto 495, Atto 565, Atto 590, Atto 647N, BODIPY, Alexa fluoro 647, Cy3B, and Cy5, for their study. They have shown their results in mitochondria, endoplasmic reticulum, endocytic vesicles, and the plasma membrane. The new strategy required only specific incubation conditions without any chemical modification or physical penetration, minimizing the damages and artifacts induced during the sample preparation. Among all, ATTO-647N dye exhibited extraordinary brightness and photostability in live cell mitochondrial labeling. Their strategy could be a future toolbox for understanding the dynamics and interactions of subcellular structures in living specimens.

Yao et al., showed the efficient application of SNAP-tag in the expansion-SRM technique using DNA oligostrands. Expansion-SRM is a new technology that anchors the dye to the hydrogel, and the dye expands with the expansion of the hydrogel so that an SRM map can be obtained under an ordinary microscope. By labeling the target protein with a first antibody and secondary antibody, the distance between the fluorescent group and the actual target protein is greatly increased. Instead of using the fluorescent proteins, which can be destroyed during sample preparation of the measurement, DNA oligostrand-linked fluorescent dye was used as an efficient probe in the expansion-SRM. The DNA oligostrand is brought to the vicinity of the target protein by specific binding technology with the SNAP. This protocol greatly reduced the error between the position of the fluorescent group and the actual target protein, and it also reduced the loss of the fluorescent group during sample preparation.

Laxman et al. showed the benefits of unnatural amino acid incorporation as protein labels for single-molecule localization microscopy (SMLM) such as stochastic optical reconstruction microscopy (STORM) and photoactivated localization microscopy (PALM). In the context of proteins, a large component of successful SMLM imaging is the choice of fluorescent probe used as a label for the protein of interest. The ideal fluorescent tag candidate for SMLM should aim for photostability during activation and deactivation cycles, be small in size, and have high levels of photon emission for better single-molecule detection by the microscope. Here, they have used protein labeling by incorporating unnatural amino acids with an attached fluorescent dye for precise localization and visualization of individual molecules. A detailed understanding and comparison of the unnatural amino acid labeling with other commonly used protein-based labeling methods, such as fluorescent proteins (FPs) or self-labeling tags like Halotag, SNAP-tags, and CLIP-tags, both highlight the benefits and shortcomings of the site-specific incorporation of unnatural amino acids coupled with fluorescent dyes in SMLM.

In another interesting article by Zhuang et al., the photobleaching imprinting technique has been utilized to reject background light and improve contrast by fully using line-scanning temporal focusing microscopy. With the removal of the background light, the method could achieve high contrast imaging both in the transparent and scattering medium.

More than 50% of total background light rejection was achieved, providing a higher signal to background ratio images of the MCF-10A samples and mouse brains. This photobleaching imprinting technology can be easily adapted to other fluorescence dyes or proteins, which may have application in studies involving relatively large and nontransparent organisms.

In this collection, the mini review, perspective, and review articles summarize the detailed information on the fluorescent nanoprobes, such lanthanide-doped upconversion nanoparticle by Dong et al., quantum, dots, carbon dots, polymer dots, and aggregation-induced emissive dots by Liu et al., and the hexagonal boron nitrides by Bradac et al. On the other hand, Cheng et al. have provided a new avenue on the biosensing interfaces with SMLM. They have given an update on the progress of using SMLM in characterizing nanostructured biointerfaces, focusing on practical aspects, recent advances, and emerging opportunities from an analytical science perspective.

In summary, the editors hope that the collection presented in this research topic, “Recent Advances in Fluorescent Probes for Super-Resolution Microscopy”, will contribute to the progress of research and development activities in the field.

